# Letter to Editor about “Non-invasive prediction of NSCLC immunotherapy efficacy and tumor microenvironment through unsupervised machine learning-driven CT radiomic subtypes: a multi-cohort study”

**DOI:** 10.1097/JS9.0000000000003104

**Published:** 2025-08-05

**Authors:** Kezhen Wang, Xin Liu, Xiuping Pan

**Affiliations:** a Department of Pharmacy, XianJu People’s Hospital, Zhejiang Southeast Campus of Zhejiang Provincial People’s Hospital, Affiliated Xianju’s Hospital, Hangzhou Medical College, Xianju, Zhejiang, China; b The Third People’s Hospital Health Care Group of Cixi, Ningbo, China

*Dear Editor,*

We read with great interest the recent article published in your journal titled *“Unsupervised learning-based identification of CT radiomic subtypes to predict immunotherapy response in non-small cell lung cancer”*^[1]^. The study presents an innovative integration of radiomics analysis with single-cell and transcriptomic sequencing, demonstrating the potential of medical imaging to non-invasively predict immunotherapy outcomes. The multi-center design and large patient cohort are commendable and add substantial value to the findings. That said, we would like to offer several suggestions from the perspectives of methodology, biological interpretation, and clinical applicability, which may help advance future research in this domain. We declare that the translation and grammar checking of this article were conducted under the guidance of TITAN^[2]^.

The study includes 1539 NSCLC patients from seven independent cohorts and performs multicenter validation, which provides a solid data foundation. However, we noticed that the primary training cohorts were predominantly composed of Asian populations, and the scRNA-seq data were derived from only a limited number of samples. This may hinder the generalizability of the model, especially to Western populations. Given that immunotherapy responses may vary with ethnicity, genetic background, and the tumor microenvironment^[3]^, we suggest that future studies incorporate more ethnically and geographically diverse datasets to enhance the universality of the findings.

The authors reported that Cluster 2 tumors were smaller, morphologically more regular, and exhibited higher immune cell infiltration, implying a potentially “immune-hot” phenotype. However, the causal links between radiographic features and immune phenotypes remain speculative. Current interpretations, such as how border spiculation or textural complexity reflects immune microenvironment status, are largely hypothetical. We recommend incorporating spatial transcriptomics or multiplex immunofluorescence to elucidate the underlying molecular mechanisms of these imaging features, which could enhance the biological interpretability of radiomic markers.

Regarding subtype classification, the authors used unsupervised K-means clustering and applied models like random forest to validate external cohorts. Although these models performed well internally (AUC >0.9), the high accuracy may partially result from similar feature distributions across datasets. To improve confidence in subtype stability, we also suggest using alternative clustering algorithms such as Nonnegative Matrix Factorization or consensus clustering to test classification robustness and assess sample overlap between different methods.

In addition, clinical imaging in real-world settings is often affected by variability in scanners, acquisition protocols, and image quality. Thus, it would be valuable to further validate model performance in real-world cohorts to evaluate robustness under more heterogeneous imaging conditions.

Lastly, the study employed overall survival (OS) and progression-free survival (PFS) as primary endpoints, with Cluster 2 patients demonstrating longer survival. Given the complexity of immunotherapy response, relying solely on traditional RECIST criteria may not fully capture the dynamic nature of immune responses. We encourage the inclusion of immunotherapy-specific criteria such as iRECIST, alongside biomarkers reflecting T cell functionality and histopathological features, to improve sensitivity and clinical relevance in response evaluation^[4]^.

In conclusion, this study offers a promising tool for the non-invasive prediction of immunotherapy efficacy in NSCLC and provides early insights into the relationship between radiomic features and the tumor immune microenvironment. We hope that future work will further refine model generalizability, validate biological mechanisms, and optimise clinical endpoints to advance the application of radiomics in precision oncology.

## Data Availability

Not applicable.
